# Preemptive Therapy Prevents Cytomegalovirus End-Organ Disease in Treatment-Naïve Patients with Advanced HIV-1 Infection in the HAART Era

**DOI:** 10.1371/journal.pone.0065348

**Published:** 2013-05-28

**Authors:** Daisuke Mizushima, Takeshi Nishijima, Hiroyuki Gatanaga, Kunihisa Tsukada, Katsuji Teruya, Yoshimi Kikuchi, Shinichi Oka

**Affiliations:** 1 AIDS Clinical Center, National Center for Global Health and Medicine, Tokyo, Japan; 2 Center for AIDS Research, Kumamoto University, Kumamoto, Japan; University of Regensburg, Germany

## Abstract

**Background:**

The efficacy of preemptive therapy against cytomegalovirus (CMV) infection remains unknown in treatment-naïve patients with advanced HIV-1 infection in the HAART era.

**Methods:**

The subjects of this single-center observation study were126 treatment-naïve HIV-1 infected patients with positive CMV viremia between January 1, 2000 and December 31, 2006. Inclusion criteria were age more than 17 years, CD4 count less than 100/μl, plasma CMV DNA positive, never having received antiretroviral therapy (ART) and no CMV end-organ disease (EOD) at first visit. The incidence of CMV-EOD was compared in patients with and without preemptive therapy against CMV-EOD. The effects of the CMV preemptive therapy were estimated in uni- and multivariate Cox hazards models.

**Results:**

CMV-EOD was diagnosed in 30 of the 96 patients of the non-preemptive therapy group (31%, 230.3 per 1000 person-years), compared with 3 of the 30 patients of the preemptive therapy group (10%, 60.9 per 1000 person-years). Univariate (HR = 0.286; 95%CI, 0.087–0.939; p = 0.039) and multivariate (adjusted HR = 0.170; 95%CI, 0.049–0.602; p = 0.005) analyses confirmed that CMV-EOD is significantly prevented by CMV preemptive therapy. Multivariate analysis showed that plasma CMV DNA level correlated significantly with CMV-EOD (per log10/ml, adjusted HR = 1.941; 95%CI, 1.266–2.975; p = 0.002). Among the 30 patients on preemptive therapy, 7 (23.3%) developed grade 3–4 leukopenia. The mortality rate was not significantly different between the two groups (p = 0.193, Log-rank test).

**Conclusions:**

The results indicate that preemptive therapy lowers the incidence of CMV-EOD by almost 25%. Preemptive therapy for treatment-naïve patients with CMV viremia is effective, although monitoring of potential treatment-related side effects is required.

## Introduction

Although the incidence of new cases of cytomegalovirus (CMV) end-organ disease (EOD) has decreased by 75%–80% with the advent of antiretroviral therapy (ART) and is currently estimated to be <6 cases per 100 person-years [1], CMV-EOD is still one of the major debilitating diseases among patients with advanced HIV infection.

CMV preemptive therapy is commonly used for patients scheduled for hematopoietic cell transplantation and solid organ transplantation, with clinical evidence of efficacy[2]–[6], however, it is not generally recommended in HIV patients [7] because of concerns regarding cost-effectiveness, risk of developing CMV resistance, side effect and the lack of a proven survival benefit [8]. A prospective trial in cooperation with Roche company to evaluate the efficacy of preemptive therapy in the pre-HAART (highly active ART) era showed significant preventive effect of oral ganciclovir (GCV) [9]. However; other studies conducted in both pre-HAART and HAART eras showed no significant effect [10], [11]. However, the above studies included patients who had previously received ART. Therefore, the efficacy of preemptive therapy against CMV infection remains unknown in treatment-naïve patients with advanced HIV-1 infection in the HAART era.

We retrospectively compared the incidence of CMV-EOD in a cohort of ART-naïve adult patients with advanced HIV infection (low CD4 count and plasma CMV-DNA-positive). One group of these patients had received CMV preemptive therapy, while the other had not received such therapy.

## Methods

### Ethics Statement

The study was approved by the Human Research Ethics Committee of National Center for Global Health and Medicine, Tokyo. All patients included in this study provided a written informed consent for their clinical and laboratory data to be used and published for research purposes. This study has been conducted according to the principles expressed in the Declaration of Helsinki.

### Study design

We performed a retrospective, single-center cohort study to elucidate the effectiveness of preemptive CMV treatment in HIV-infected patients with positive CMV viral load in the prevention of CMV-EOD. The study was conducted at the National Center for Global Health and Medicine, Tokyo, one of the largest clinics for patients with HIV infection in Japan, with more than 2,700 registered patients as of December 2006. The study population comprised treatment-naïve HIV infected patients aged more than 17 years, with CD4 count less than 100/μl and positive plasma CMV DNA viral load, who presented for the first time at our hospital between January 1, 2000 and December 31, 2006. Those with CMV-EOD at presentation and those with <3 months of follow-up were excluded. The follow-up period was 2 years from the initial visit.

### Definition of CMV-EOD and CMV preemptive therapy

CMV-EOD was diagnosed according to standardized ACTG criteria (see Table S1) [11]. CMV retinitis was routinely screened for by dilated indirect ophthalmoscopy at both the first visit to the hospital and a few months after the commencement of ART. Other evaluations, such as endoscopy and bronchoscopy, were carried out in response to the symptoms and clinical condition. The diagnosis of CMV-EOD was established by at least two experts from our hospital.

CMV preemptive therapy was prescribed based on the clinician's assessment. CMV preemptive therapy was provided at our institution for patients with plasma CMV DNA of >5000 copies/ml. For patients with plasma CMV DNA of >3000 but less than 5000 copies/ml, the decision to initiate preemptive therapy was left to the attending physician, taking into consideration the overall clinical condition, such as subsequent rise in plasma CMV DNA and/or use of immunosuppressants, such as steroids and chemotherapeutic agents. Ganciclovir (GCV) and valganciclovir (VGCV) were the most commonly used agents, followed by foscarnet (FOS). The choice of induction (intravenous GCV 5 mg/kg every 12 hours, oral VGCV900 mg twice a day or intravenous FOS 90 mg/kg every 12 hours) or maintenance dose (intravenous GCV 5 mg/kg every 24 hours, oral VGCV 900 mg a day or intravenous FOS 90 mg/kg every 24 hours) was based on the clinical condition, such as the level of plasma CMV DNA or state of immunosuppression. The duration of therapy varied across individuals. CMV preemptive therapy was defined as at least a 7-day treatment with agents effective against CMV. The normal course of CMV preemptive therapy was 2 weeks of GCV induction dose followed by VGCV or GCV maintenance dose until plasma CMV DNA became negative. Patients were retreated based on clinicians' decision under some conditions with high risks for CMV-EOD as described above, if plasma CMV DNA became positive again after preemptive therapy.

### Measurements

Plasma CMV DNA was measured using real-time PCR with a lower limit of detection of 200 copies/mL(CMV geniQ, Bio Medical Laboratory, Inc., Tokyo, Japan). Plasma CMV DNA was measured routinely at the first visit in patients with CD4 count of <100/μl, and re-examined every week or monthly, according to the level of plasma CMV DNA viral load or immune status and at the discretion of the attending physician.

In this study, the primary exposure variable was CMV preemptive therapy over no CMV preemptive therapy. The potential risk factors for CMV-EOD were determined based on previous studies [12]–[18], and included basic demographics and laboratory data, including age, sex, CD4 cell count, HIV viral load, plasma CMV DNA, and presence or absence of other medical conditions (concurrent use of steroids, concurrent chemotherapy and concurrent AIDS-defining diseases). For each patient, data on or closest to the day of the first visit to our hospital were retrieved for analysis.

### Statistical analysis

Categorical and continuous baseline demographics and laboratory data were analyzed using Pearson's chi-square test and Student's t-test, respectively. The time from the first visit to our hospital to the development of CMV-EOD was analyzed by the Kaplan Meier method for patients on CMV preemptive therapy and no CMV preemptive therapy, and the log-rank test was used to determine the statistical significance. Censored cases represented those who died, dropped out, or were referred to other facilities before the end of follow-up period. The Cox proportional hazards regression analysis was used to estimate the impact of CMV preemptive therapy on the incidence of CMV-EOD. The impact of basic demographics, baseline laboratory data, and other medical conditions was also estimated with univariate Cox proportional hazards regression.

To estimate the unbiased prognostic impact of CMV preemptive therapy, we used three models based on multivariate Cox proportional hazards regression analysis. Model 1 was the aforementioned univariate analysis for CMV preemptive therapy. Model 2 included age and sex, plus Model 1, in order to adjust for basic characteristics. In Model 3, we added variables with significant relation to CMV-EOD by univariate analysis or assumed as risk factor(s) for CMV-EOD in the literature[12]–[20] (e.g., CD4 count per 1/μl decrement, HIV viral load per log10/ml, CMVDNA viral load per log10/ml, concurrent steroid use, concurrent chemotherapy and concurrent AIDS defining disease). Statistical significance was set at two-sided *p* values <0.05. We used hazard ratios (HRs) and 95% confidence intervals (95%CIs) to estimate the impact of each variable on CMV-EOD. All statistical analyses were performed with The Statistical Package for Social Sciences ver. 17.0 (SPSS, Chicago, IL).

## Results

**Figure 1:**
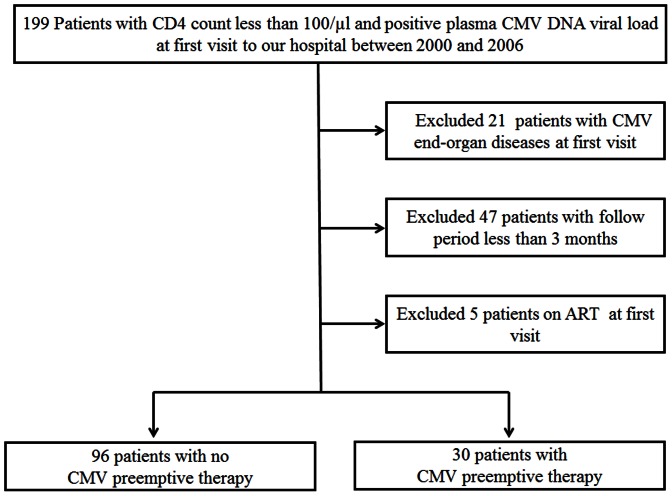
Flow chart of inclusion and exclusion criteria. Of the 199 subjects, 73 were excluded and the remaining 126 were included in the study. The latter group was divided into the preemptive therapy group (n = 30) and the non-therapy group (n = 96).

Of the 199 HIV-infected patients with CD4 count <100/μl and positive plasma CMV DNA viral load referred to our hospital between January 1, 2000 and December 31, 2006, 126 patients were recruited in the study. Of these, 96 patients received CMV preemptive therapy while 30 did not (Figure 1). Table 1 lists the demographics, laboratory data, and medical conditions of the study population at baseline. The majority of the study population were males, East Asians, and relatively young (median: 42 years). There were no differences in baseline CD4 count (p = 0.595) and HIV viral load (p = 0.628) between the two groups. Patients of the CMV preemptive therapy group had higher plasma CMV DNA viral load (p<0.001), more likely to have developed AIDS defining diseases (p = 0.042), and tended to have been treated concurrently with steroids (p = 0.009), compared with the non-CMV preemptive group. There were no significant differences in the use of chemotherapy (p = 1.000) and in time to initiation of ART since study entry (p = 0.393, Table 1) between the two groups.

**Table 1:** Baseline demographics and laboratory data of patients who did and did not receive CMV preemptive therapy.

	Non-preemptive therapy (n = 96)	Preemptive therapy (n = 30)	P value
Sex (male), n (%)	88 (91.7)	29 (96.7)	0.685
Median (range) age	41 (24–76)	44 (25–66)	0.729
Ethnicity, n (%)			
East Asians	86 (89.5)	29 (96.7)	
Southeast Asian	5 (5.2)	0 (0.0)	
Black	3 (3.1)	0 (0.0)	
White	2 (2.1)	1 (3.3)	
Median (range) CD4 count (/μl)	28.0 (0–97)	35.5 (3–87)	0.595
Median (range) HIV RNA viral load (log10/ml)	5.3 (3–6)	5.35 (4–7)	0.628
Median (range) CMVDNA viral load (log10/ml)	3.0 (2–5)	4.3 (2–5)	<0.001
Concurrent AIDS, n (%)	78 (81.3)	29 (96.7)	0.042
Steroid use, n (%)	38 (39.6)	20 (66.7)	0.009
Chemotherapy, n (%)	9 (9.4)	2 (6.7)	1.000
Median (range) time (days) to ART*	66 (2–399)	59 (13–158)	0.393
Median (range) follow-up (days)	730 (14–730)	730 (25–730)	0.064

* 11 missing values.

Categorical and continuous variables were analyzed using Pearson's chi-square test and Student's t-test, respectively.

**Figure 2:**
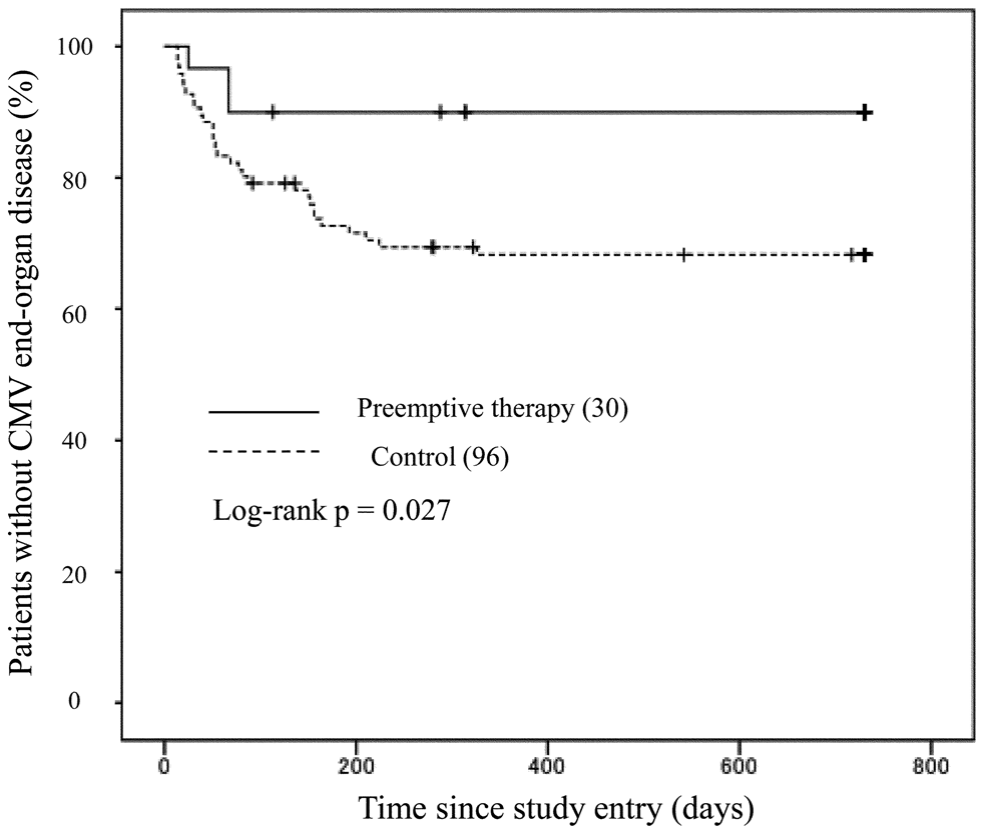
Kaplan-Meier curve showing the time to development of cytomegalovirus (CMV)- end-organ disease (EOD) in the preemptive and non-preemptive therapy groups. Compared to patients on CMV preemptive therapy, those who did not receive preemptive therapy were more likely to develop CMV-EOD (p = 0.027, Log-rank test).

During the follow-up period, CMV-EOD occurred in 3 (10.0%) patients of the preemptive therapy group and 30 (31.3%) of the non-preemptive therapy group, with an estimated incidence of 60.9 and 230.3 per 1000 person-years, respectively. Figure 2 depicts the time from the first visit to our hospital to the development of CMV-EOD by Kaplan Meier method in the two groups. The incidence of new cases of CMV-EOD was significantly higher in the non-preemptive therapy group, compared with the preemptive therapy group (p = 0.027, Log-rank test). The median time from the first visit to the diagnosis of CMV-EOD was 67 days (range, 25–67) for the preemptive therapy group, and 54 days (range, 14–326 days) for the non-preemptive therapy group.

Univariate analysis showed a significant relationship between CMV preemptive therapy and low incidence of CMV-EOD (HR = 0.286; 95%CI, 0.087–0.939; p = 0.039) (Table 2). On the other hand, high CMV viral load and HIV viral load tended to be associated with CMV-EOD, while old age, low baseline CD4 count, use of steroids, chemotherapy, and concurrent AIDS defining diseases were not associated with CMV-EOD. Multivariate analysis identified CMV preemptive therapy as a significant preventive factor against CMV-EOD after adjustment for age and sex (Model 2; adjusted HR = 0.289; 95%CI, 0.088–0.949; p = 0.041, Table 3), and after adjustment for other risk factors (Model 3; adjusted HR = 0.172; 95%CI, 0.049–0.602; p = 0.005, Table 3). In addition, multivariate analysis showed that high CMV viral load correlated significantly with CMV-EOD (Model 3; adjusted HR = 1.941; 95%CI, 1.266–2.975; p = 0.002, Table 3).

**Table 2:** Results of univariate analysis to estimate the risk of various factors in inducing CMV end-organ disease.

	Hazard ratio	95% CI	P value
CMV preemptive therapy	0.286	0.087–0.939	0.039
Female	1.284	0.392–4.209	0.680
Age per 1 year	0.982	0.951–1.013	0.240
CD4 count per 1/μl decrement	1.001	0.989–1.013	0.867
HIV viral load per log10/ml	1.875	0.905–3.884	0.091
CMV viral load per log10/ml	1.450	0.984–2.136	0.060
Use of steroid	0.716	0.356–1.439	0.348
Chemotherapy	1.390	0.488–3.955	0.537
Concurrent AIDS	0.703	0.290–1.704	0.436

CI: confidence interval

The Cox proportional hazards regression analysis was used.

**Table 3:** Results of multivariate analysis to estimate the preventive effect of CMV preemptive therapy against CMV end-organ disease.

	Model 1 Crude		Model 2 Adjusted		Model 3 Adjusted	
	HR	95% CI	HR	95%CI	HR	95%CI
CMV preemptive therapy^†^	0.286	0.087–0.939	0.289	0.088–0.949	0.172	0.049–0.602
Age			0.982	0.952–1.014	0.990	0.958–1.022
Female			1.033	0.310–3.441	0.988	0.267–3.653
CD4 count per 1/μl decrement					0.995	0.983–1.008
HIV viral load per log10/ml					2.217	0.912–5.393
CMV viral load per log10/ml*					1.941	1.266–2.975
Use of steroid					0.664	0.288–1.534
Chemotherapy					1.668	0.540–5.151
Concurrent AIDS					0.930	0.337–2.569

* P<0.05 in Model 3

HR: hazard ratio, CI: confidence interval

The Cox proportional hazards regression analysis was used.

Variables with significant difference by univariate analysis or assumed as risk factors for CMV-EOD in the literature were included in model 3.

Of the 33 patients with CMV-EOD, 22 (66.7%) developed CMV retinitis, 4 (12.1%) developed esophagitis, 3 (9.1%) developed gastroduodenitis, 6 (18.2%) developed colitis and 1 (3.0%) developed pneumonitis. All 3 patients with CMV-EOD of the preemptive therapy group developed retinitis (Table 4).

**Table 4:** Details of CMV end-organ disease.

CMV-EOD	n (%)	Time to development (days)	Non-preemptive therapy group	Preemptive therapy group
Retinitis	22* (61.1%)	72 (14–326)	19* (57.6%)	3 (100%)
Esophagitis	4* (11.1%)	116.5(69–164)	4* (12.1%)	0
Gastroenteritis	3* (8.3%)	19 (14–40)	3* (9.1%)	0
Colitis	6* (16.7%)	40.5 (15–55)	6* (18.2%)	0
Pneumonitis	1 (2.8%)	31 (31–31)	1 (3.0%)	0
Total	36* (100%)	55 (14–326)	33* (100%)	3 (100%)

* Three patients of the non-preemptive therapy group had multiple CMV-EOD; one with retinitis plus esophagitis, one with retinitis plus gastroenteritis and the other with retinitis plus colitis.

**Figure 3:**
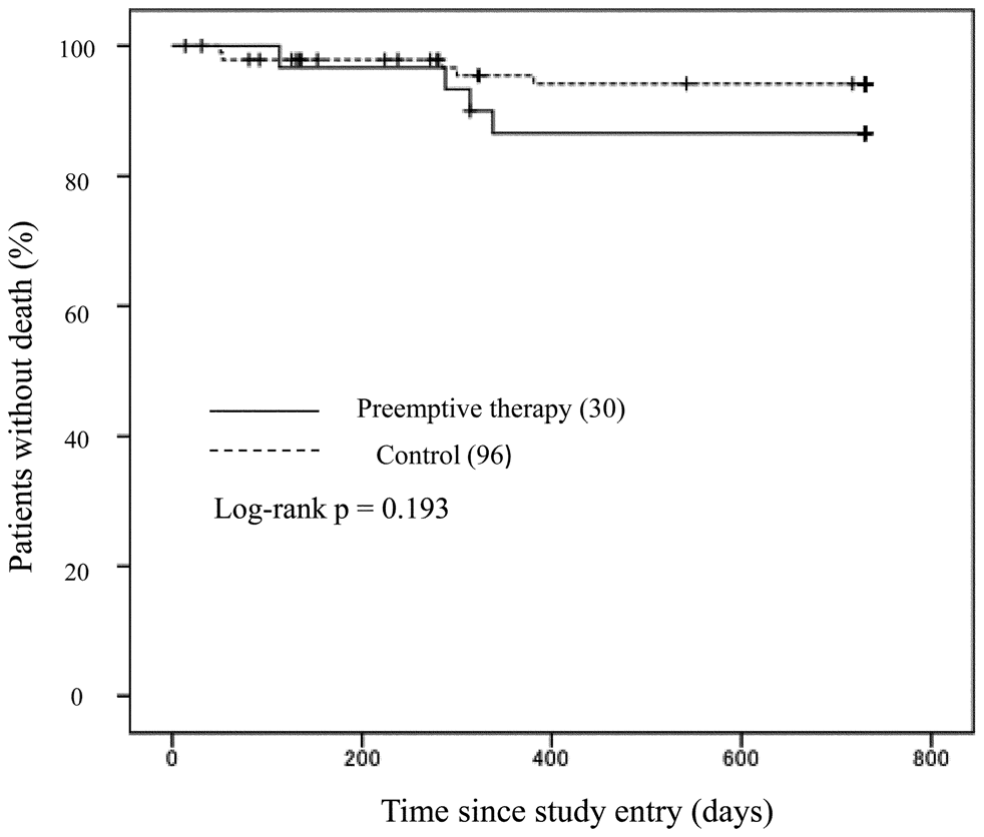
Kaplan-Meier curve showing the time to death in the preemptive and non-preemptive therapy groups. There was no significant difference in the survival rate between the two groups (p = 0.193, Log-rank test).

Of 30 patients who received preemptive therapy, 20 (66.7%) received an induction dose of GCV, and 7 patients (23.3%) received maintenance dose. The remaining agents used for preemptive therapy were an induction dose of VGCV, a maintenance dose of FOS and an induction dose of cidofovir. The duration of the preemptive therapy varied between 7 days and 2 months. The following side effects were noted in patients on CMV preemptive therapy: grade 3/4 leukopenia (n = 7, 23.3%) and grade 2 hypercreatininemia (n = 1, 3.3%). Both side effects developed during the use of GCV. Five patients (5.2%) of the non-preemptive therapy group and 4 patients (13.3%) of the preemptive therapy group died during the study period. Of the former group, 3 deaths were due to opportunistic infections (cryptococcus meningitis, non-tuberculous mycobacterial infection and *Pneumocystis jiroveci* pneumonia), 1 due to bacterial infection (sepsis), and 1 due to suicide. Of the latter group, 2 deaths were due to opportunistic infections (malignant lymphoma and *P. jiroveci* pneumonia) and 2 due to bacterial infection (bacterial pneumonias). Deaths and bacterial infections related to preemptive therapy were not observed in our study. The mortality rate was not significantly different between the two groups (p = 0.193, Log-rank test, Figure 3).

## Discussion

The results of this observational cohort of treatment-naïve HIV-infected patients with positive plasma CMV DNA showed a significantly lower incidence of CMV-EOD by one-fourths in the CMV preemptive therapy group than in the non-preemptive therapy group, over the 2-year observation period. This finding was significant, despite higher risk for CMV-EOD in the preemptive therapy group, such as higher plasma CMV DNA, higher prevalence of concurrent AIDS defining diseases and more concurrent steroid use, compared with the other group. Univariate and multivariate analyses identified anti-CMV preemptive therapy as a significant preventive factor against CMV-EOD.

Our study is the first to illustrate the significance of anti-CMV preemptive therapy in treatment- naïve HIV-infected patients with CMV viremia and CD4 count less than 100/μl in the HAART era. The hazard ratio of development of CMV-EOD decreased by 82.8% following preemptive therapy, compared with no preemptive therapy, even after adjustment for plasma CMV DNA viral load and other factors. The current guidelines do not generally recommend anti-CMV preemptive therapy although this is based on sparse evidence, such as cost effectiveness, CMV resistance, and drug side effects [7]. However, our study suggests that preemptive therapy is a feasible option, if the effective target of preemptive therapy could be selected. Furthermore, the study confirmed that plasma CMV DNA, a known risk factor for CMV-EOD [12]–[18], was a significant independent risk factor.

A few prospective clinical trials investigated the efficacy of preemptive therapy in both the pre-HAART era and HAART era. In these studies, oral GCV at 1000 mg thrice daily was used in the pre-HAART era regimen [9], [10] while VGCV at 900 mg twice daily was the regimen used in the HAART era [11]. The patients investigated in the above three studies were HIV-treatment-experienced patients. One study in the pre-HAART era reported the efficacy of preemptive therapy in patients with CD4 count<50 µl [9], while the other studies showed no significant preventive effect [10], [11]. In the ACTG A5030 study, the prospective clinical trial in the HAART era, which evaluated the efficacy of oral VGCV 900 mg twice a day for 3 weeks among HIV-infected patients with CD4 count <100 cells/mm^3^, plasma HIV RNA >400 copies/mL, plasma CMV viremia and on stable or no HAART, the authors reported a low incidence of CMV-EOD among subjects both with and without preemptive therapy [11]. The authors attributed the low incidence to improvement of immune function induced by potent ART. Actually, in that study [11], the number of patients who had received ART at study entry was about 80% of the total. In contrast, the subjects of our study were all treatment-naïve patients and possibly at higher risk for CMV-EOD compared to those enrolled in the ACTG A5030. Thus, the use of CMV preemptive therapy reported in our study under the clinical scenario of poor immune status without ART at study entry resulted in better outcome than in previous studies. In our study, there was no significant difference in the timing of ART between the two treatment groups. Although our study did not include the time to the initiation of ART as a variable in uni- and multivariate analysis because the values for 11 cases were missing, multivariate analysis with the time to the initiation of ART together with other variables similarly identified preemptive therapy as a significant preventive factor (adjusted HR = 0.235; 95%CI, 0.064–0.868; p = 0.030).

The survival benefits of CMV preemptive therapy were controversial in previous prospective clinical trials. One study suggested the survival benefits of 3 g/day oral GCV preemptive therapy [9], while other studies showed no evidence of the survival benefit [10]. On the other hand, two prospective cohort studies in the HAART era showed the relation between CMV viremia and high mortality [21] and suggested the benefit of CMV therapy [22], whereas our results showed no significant difference in mortality rate between the two groups. The reason for this discrepancy could be attributed to low mortality rate, small sample size and the disproportionally high risk of the therapy group in our study. The mortality rate (5.0 deaths per 100 person-years) in our study was similar to that in a study conducted in the HAART era (5.7 deaths per 100 person-years)[19] and was considerably lower than in studies from the pre-HAART era. Since the mortality rate has markedly decreased in advanced HIV infected patients following the introduction of potent ART in the HAART era [23], [24], not only the survival benefit but also quality of life, such as improvement of eye function, should be emphasized in the future.

The side effects of preemptive therapy have also been of concern [25]. Our findings showed the development of grade 3 to 4 leukocytopenia in 23.3% of the patients who received intravenous GCV, and was the major side effect of preemptive therapy. Some patients who developed leukocytopenia required treatment with granulocyte colony-stimulating factor (G-CSF) and showed complete recovery. Thus; careful follow-up of patients on preemptive therapy is necessary. For these reasons, preemptive therapy might place patients at greater risk in resource-limited setting, where close monitoring is difficult and the risk of bacterial infection is high. It is noteworthy, however, that death and bacterial infection related to preemptive therapy were not observed in our study.

The present study has several limitations. Due to its retrospective nature, it was not possible to control the baseline characteristics of the enrolled patients. However, patients with potential risk for CMV-EOD, such as those with high plasma CMV DNA, high concurrent AIDS and high steroid use, were more likely prescribed the preemptive therapy. It is noteworthy that the incidence of CMV-EOD was significantly lower in the preemptive therapy group despite this adverse environment.

Second, the criteria for treatment, choice of drugs and duration of CMV preemptive therapy were not rigidly controlled in the present study. Thus, it was difficult to determine which anti-CMV agent with what dosage is optimal for preemptive therapy. In the present study, about 90% of patients received induction dose or maintenance dose of GCV since the majority of patients of the preemptive therapy group were in-patients. Further prospective study is required to optimize effective preemptive therapy, including oral VGCV.

Third, CMV-EOD, especially enteritis, could have been overlooked at study entry since routine endoscopic screening was not performed, compared with screening for retinitis at the first visit. However, patients with abdominal pain were subjected to stool examination for occult blood, since the definition of CMV enteritis includes abdominal pain, and those with positive tests were subsequently considered for endoscopy. Thus, the possibility of latent CMV enteritis at study entry does not seem to have affected the results of the present study.

In conclusion, the present study demonstrated a lower incidence of CMV-EOD following CMV preemptive therapy by one-fourth, compared with no preemptive therapy, in treatment-naïve patients with CMV viremia. High plasma CMV DNA was identified as an independent risk for CMV-EOD. Further studies are warranted to elucidate the efficacy, safety and cost-effectiveness of anti-CMV preemptive therapy in HIV infected patients at high risk for EOD.

## Supporting Information

Table S1 **Definitions of CMV end-organ diseases used in this study.**
(DOCX)
