# Supplementary material for: Preemptive Therapy Prevents Cytomegalovirus End-Organ Disease in Treatment-Naïve Patients with Advanced HIV-1 Infection in the HAART Era
Source: PLoS One. 2013 May 28;8(5):e65348. doi: 10.1371/journal.pone.0065348 (PMC3665626; doi:10.1371/journal.pone.0065348)
Supplement: Table S1 — Definitions of CMV end-organ diseases used in this study. (DOCX) [file pone.0065348.s001.docx]

Table S1. Definitions of CMV end-organ disease used in this study

| CMV diseases | Definition |
| --- | --- |
| Confirmed CMV retinitis | Typical lesions including white areas with or without hemorrhages and/or gray-white areas of retinal necrosis with or without hemorrhages. Lesion(s) has/have irregular, dry-appearing, granular border, with little or no overlying vitreous inflammation. Must be diagnosed by an experienced ophthalmologist using indirect ophthalmoscopy and documented by retinal photography that can be independently verified. |
| Probable CMV retinitis | Typical lesions including white areas with or without hemorrhages and/or gray-white areas of retinal necrosis with or without hemorrhages. Lesion(s) has/have irregular, dry-appearing, granular border, with little or no overlying vitreous inflammation. Must be diagnosed by an experienced ophthalmologist using indirect ophthalmoscopy but is not documented by retinal photographs. |
| Confirmed CMV esophagitis | Presence of at least one of the following: retrosternal pain or odynophagia (pain on swallowing). **AND** Endoscopically-confirmed mucosal erythema, erosion, or ulceration. **AND** Tissue biopsy demonstrating CMV by antigen or characteristic cytopathic changes. |
| Probable CMV esophagitis | Presence of at least one of the following: retrosternal pain or odynophagia (pain on swallowing). **AND** Endoscopically-confirmed mucosal erythema, erosion, or ulceration. **AND** CMV is isolated from the lesion **AND** Anti-CMV therapy initiated or recommended. |
| Confirmed CMV gastroenteritis | Presence of abdominal pain. **AND** Endoscopically-confirmed mucosal erythema, erosion, or ulceration. **AND** Tissue biopsy with CMV by antigen or characteristic cytopathic changes. |
| Probable CMV gastroenteritis | Presence of abdominal pain. **AND** Endoscopically-confirmed mucosal erythema, erosion, or ulceration. **AND** CMV is isolated from the lesion **AND** Anti-CMV therapy initiated or recommended. |
| Confirmed CMV colitis | Presence of at least one of the following: abdominal pain or diarrhea (typically in small volume and associated with mucus and blood). **AND** Colonoscopically-, sigmoidoscopically-, or endoscopically-confirmed mucosal erythema, erosion, or ulceration. **AND** Tissue biopsy demonstrating CMV by antigen or characteristic cytopathic changes. |
| Probable CMV colitis | Presence of at least one of the following symptoms: abdominal pain or diarrhea (typically in small volume and associated with mucus and blood). **AND** Colonoscopically-, sigmoidoscopically-, or endoscopically-confirmed mucosal erythema, erosion, or ulceration. **AND** CMV is isolated from the lesion **AND** Anti-CMV therapy initiated or recommended. |
| Confirmed CMV proctitis | Presence of rectal pain, often associated with tenesmus, mucus, and blood. **AND** Colonoscopically-, sigmoidoscopically-, or endoscopically-confirmed mucosal erythema, erosion, or ulceration. **AND** Tissue biopsy demonstrating CMV by antigen or characteristic cytopathic changes. |
| Probable CMV proctitis | Presence of rectal pain, often associated with tenesmus, mucus, and blood. **AND** Colonoscopically-, sigmoidoscopically-, or endoscopically-confirmed mucosal erythema, erosion, or ulceration. **AND** CMV is isolated from the lesion **AND** Anti-CMV therapy initiated or recommended.. |
| Confirmed CMV pneumonitis | CMV pneumonitis: Hypoxemia and infiltrates on chest X-ray or CT/MRI scan. **AND** tissue biopsy or cells obtained by BAL demonstrating CMV antigen or characteristic cytopathic changes. **AND** no other pathogens identified by routine testing or signs/symptoms persist or recur after treatment of copathogens. |
| Probable CMV pneumonitis | Hypoxemia and infiltrates on chest X-ray or CT/MRI scan. **AND** positive MV culture or detection of CMV antigen from fluid obtained by BAL. **AND** no other pathogens identified by routine testing or signs/symptoms persist or recur after treatment of copathogens. **AND** Anti-CMV therapy initiated or recommended. |
| Confirmed CMV encephalitis | Progressive changes in mental status, delirium, rapidly progressive cognitive impairment, or signs and symptoms of brain stem injury **AND** detection of viral nucleic acids (e.g., PCR) in CSF CMV culture positive or brain biopsy demonstrating CMV by antigen, detection of viral nucleic acids (e.g., PCR), or characteristic cytopathic changes. |
| Probable CMV encephalitis | Progressive changes in mental status, delirium, rapidly progressive cognitive impairment, or signs and symptoms of brain stem injury **AND** MRI or contrast CT scan performed which: a) excludes toxoplasmosis, lymphoma, PML, or other intracranial process, and b) demonstrates periventricular inflammation or meningeal enhancement. **AND** other etiologies ruled out. **AND** CMV-EOD (e.g., retinitis, colitis) present. **AND** specific therapy initiated, changed, or recommended. |
| Confirmed other syndromes | |
| Hepatitis or cholangitis | ALP or ALT significantly elevated above the patient's baseline values **AND** tissue biopsy demonstrating CMV by antigen or characteristic cytopathic changes. |
| Radiculomyelopathy | Clinical presentation compatible with CMV-EOD, including all of the following: **a.** Decreased lower extremity strength and reflexes or syndrome consistent with a cord lesion presently subacutely (over days to weeks), **b.** Myelography or MRI reveals no mass lesions but thickening of lower spinal nerve roots, **c.** CMV-positive culture in CSF **OR** detection of CMV viral nucleic acids (e.g., PCR) in CSF. |
| Confirmed cutaneous ulcers | Direct visualization of oral or vulvovaginal or perianal ulcers. **AND** CMV culture of lesion or histopathological demonstration of typical CMV cytopathology on biopsy of lesion. |
